# Effectively Improve the Astaxanthin Production by Combined Additives Regulating Different Metabolic Nodes in *Phaffia rhodozyma*

**DOI:** 10.3389/fbioe.2021.812309

**Published:** 2022-01-17

**Authors:** Zhipeng Li, Haoyi Yang, Chenhua Zheng, Xiping Du, Hui Ni, Ning He, Liang Yang, Li You, Yanbing Zhu, Lijun Li

**Affiliations:** ^1^ College of Food and Biology Engineering, Jimei University, Xiamen, China; ^2^ Fujian Provincial Key Laboratory of Food Microbiology and Enzyme Engineering Technology, Xiamen, China; ^3^ Research Center of Food Biotechnology of Xiamen City, Xiamen, China; ^4^ Department of Chemical and Biochemical Engineering, College of Chemistry and Chemical Engineering, Xiamen University, Xiamen, China

**Keywords:** *phaffia rhodozyma*, astaxanthin, metabolic pathways, metabolic regulators, combined additives

## Abstract

Astaxanthin is an important natural resource that is widely found in marine environments. Metabolic regulation is an effective method for improving astaxanthin production in *Phaffia rhodozyma*. Most studies have focused on single regulators, which have limited effects. In this study, 16 metabolic regulators were screened to improve astaxanthin production in high-yield and wild-type strains. Fluconazol and glutamic acid increased astaxanthin volumetric yield in MVP14 by 25.8 and 30.9%, respectively, while ethanol increased astaxanthin volumetric yield in DSM626, 29.3%. Furthermore, six additives that inhibit the competing pathways and promote the main pathway for astaxanthin synthesis were selected for combination treatment. We found that the optimal combination was penicillin, ethanol, triclosan, and fluconazol, which increased astaxanthin cell yield by 51%. Therefore, we suggest that simultaneously promoting the master pathways (mevalonate) and inhibiting competing pathways (fatty acid synthesis and ergosterol) is the best strategy to improve astaxanthin cell yield. Moreover, regulators of the biomass pathway should be avoided to improve cell yield. This study provides a technical basis for the utilisation of astaxanthin in *P. rhodozyma*.

**GRAPHICAL ABSTRACT:**
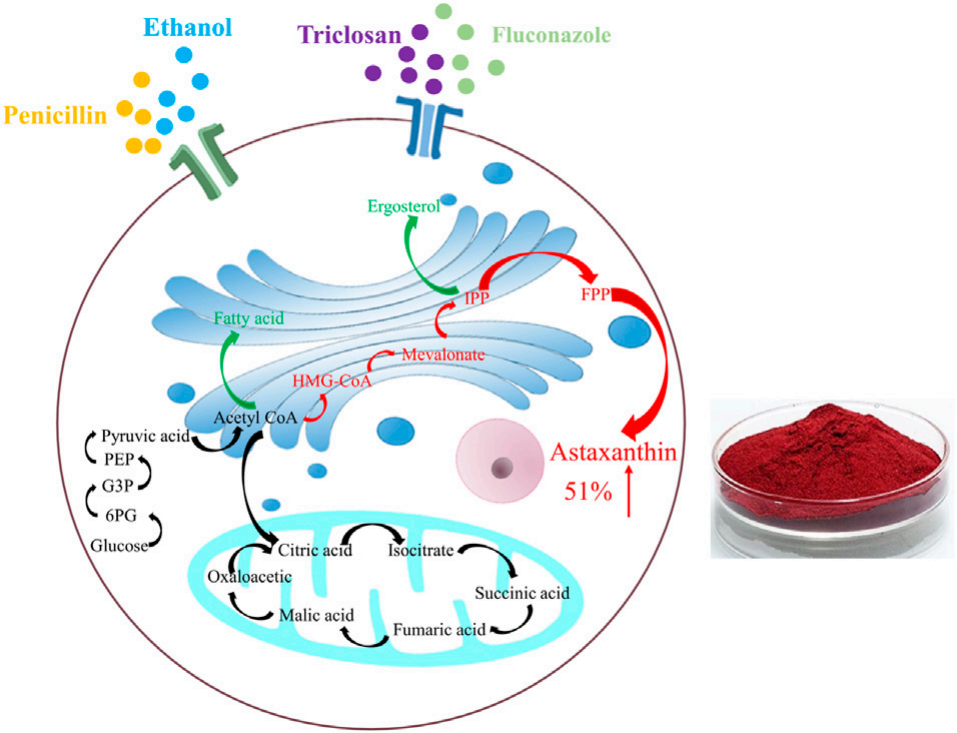


After adding penicillin, ethanol, triclosan and fluconazol, the changes happened in the metabolic pathways of *P. rhodozyma* cells. Red represents up-regulation of the substance or pathway, and green represents down-regulation.

## Introduction

Astaxanthin (3,3-dihydroxy-beta,beta-carotene-4,4′-dione) is a ketocarotenoid with antioxidant, anti-inflammatory, anti-aging, anti-tumour, and immune-enhancing properties (Mimoun-Benarroch et al., 2018; Lai et al., 2021; Zhou et al., 2021). It is widely present in marine environments and plays an important role in the survival of various marine organisms (Schmidt et al., 2011). The main natural sources of astaxanthin are *Phaffia rhodozyma* and *Haematococcus pluvialis* (Schmidt et al., 2011). *P. rhodozyma*, a basidiomycete, is an important biological source of astaxanthin (Liu et al., 2021; Torres-Haro et al., 2021). Compared with other strains, the fermentation of astaxanthin by *P. rhodozyma* offers many advantages, such as simple culture requirements and ease of production (Stoklosa et al., 2019). The astaxanthin yield of wild *P. rhodozyma* is low at only 0.17–1.73 mg/g dry cell (Domínguez-Bocanegra, 2007; Batghare et al., 2018), and cannot meet industrial production requirements (Kothari et al., 2019).

Metabolic regulation is an important method to improve microbial productivity (Lai et al., 2021). The production of astaxanthin in *P. rhodozyma* can be affected by many complex metabolic pathways, such as the hexose monophosphate (HMP) pathway, Embden-Meyerhof-Parnas (EMP) pathway, mevalonate (MVA) pathway, tricarboxylic acid (TCA) cycle, fatty acid synthesis (FAS) pathway, and ergosterol synthesis pathway (Pan et al., 2020; Wan et al., 2021; Zhang et al., 2021). Among these pathways, HMP, EMP, and MVA pathways are necessary in the synthesis of astaxanthin (Lan et al., 2002; Wang et al., 2012; Wushensky et al., 2018), whereas TCA, FAS, and ergosterol synthesis pathways are competing pathways (Sanglard et al., 1998; Cheng et al., 2016; Wang, L. et al., 2019). The production of carotenoids, including astaxanthin, can be increased by adding metabolic regulators which affect these metabolic pathways. Gu et al. (1997) increased the carotenoid production in *P. rhodozyma* by 0.84 mg/g by adding 0.2% ethanol to the medium. Miao et al. (2011) increased astaxanthin production in *P. rhodozyma* by 0.01 and 0.2 mg/g in 96 h by adding triclosan and fluconazol, respectively. Chen et al. (2021) reported that pyruvate can increase astaxanthin content in *Chromochloris zofingiensis* by 25%. It can be seen that single modulator is effective but its impact is very limited.

Therefore, systematic screening of single regulators could help analyse their regulatory functions and effects on metabolic pathways. Previous studies have shown that inhibiting competing pathways can remarkably increase the yield of target products. Lee et al. (2018) reported that the elimination of L-valine and L-isoleucine can enhance isobutanol production in *Saccharomyces cerevisiae*. Similarly, the promotion and regulation of synthetic pathways can also achieve positive results; for example, light can promote carotenoid accumulation (Sun et al., 2019). Therefore, we speculated that biosynthetic and competing metabolic pathways could be regulated simultaneously to achieve a more optimal effect.

In this study, we explored the effects of 15 related additives on the above mentioned six pathways. These additives are known to exert promotive or competitive effects on astaxanthin production. We further optimised a combination of these additives. Furthermore, the effects of different metabolic pathways on astaxanthin synthesis were systematically analysed to investigate the mechanism of astaxanthin synthesis. This study could provide technical support to improve the astaxanthin industry and provide a theoretical basis for the study of astaxanthin metabolism.

## Material and Methods

### Strains and Culture Conditions

*P. rhodozyma* JMU-MVP14 was preserved by Fujian Provincial Key Laboratory of Food Microbiology and Enzyme Engineering Technology, Jimei University. The astaxanthin-overproducing mutant *P. rhodozyma* JMU-MVP14 was established through ethyl methyl sulfonate mutagenesis from JMU-VDL668. *P. rhodozyma* DSM5626 (ATCC 24202) was purchased from the German Collection of Microorganisms and Cell Cultures (Germany).

Two generations of cultivation were performed in yeast extract–peptone–dextrose (YPD) medium. Then, each strain was fermented in another YPD medium. The YPD medium, which was composed of 10 g/L yeast powder, 20 g/L peptone, and 20 g/L glucose and had a pH of 6.0, was sterilized at 121°C for 30 min. Add 30 ml of fresh medium to the 250 ml Erlenmeyer flask and inoculate with 3% of the inoculum. The first-generation cultured 48h, the second-generation cultured 24 h. The culture temperature was 22°C, and the rotational speed of shaker was 200 r/min.

### Addition Method

Different regulators, namely, pyruvic acid, glutamic acid, sodium fluoride, α-ketoglutarate, simvastatin, N-methyl morpholine, malonic acid, nicotine, citric acid, sodium gluconate, and sodium phosphate, were added before sterilization. Ethanol was added during inoculation, penicillin was added at 36 h of fermentation, and fluconazol and triclosan were added according to different conditions. The concentrations and time of the regulations are shown in Table 1.

**TABLE 1:** Adding method of metabolic regulators.

Metabolic regulators	Added concentration	Time of adding	Pathway	References
Glutamic acid	3 g/L	Before sterilization	Promoting HMP	An, 2001; Lan et al., 2002
Sodium fluoride	1.5 mg/L	Before sterilization	Promoting HMP	GumiŃska and Sterkowicz, 1975
Sodium phosphate	1.5 mg/L	Before sterilization	Inhibiting HMP	Liu et al., 2022
Sodium gluconate	1 g/L	Before sterilization	Promoting EMP	Wushensky et al. (2018)
Citric acid	2 g/L	Before sterilization	Inhibiting EMP	Li et al. (2012)
Penicillin	1 mg/L	36th h of fermentation	Promoting MVA	Wang et al. (2012)
Malonic acid	0.5 mg/L	Before sterilization	Inhibiting TCA Cycle	Wang, L. et al. (2019)
Pyruvic acid	2 g/L	Before sterilization	Promoting TCA Cycle	Chen et al. (2021)
α-ketoglutaric acid	0.5 g/L	Before sterilization	Promoting Accumulation of Acetyl CoA	Li et al. (2012)
Nicotine	0.04%	Before sterilization	Inhibiting dehydrogenase Activity	Fazeli et al. (2009)
Triclosan	5 mg/L	48th h of fermentation	Inhibiting FAS Pathway	Cheng et al. (2016)
Fluconazol	120 mg/L	48th h of fermentation	Inhibiting Ergosterol Synthesis Pathway	Sanglard et al. (1998)
Ethanol	1 g/L	0 h of fermentation	Promoting HMGR Enzyme Activity	Gu et al. (1997)
Simvastatin	0.02 g/L	Before sterilization	Inhibiting HMGR Enzyme Activity	Susilowati et al. (2020)
N-methyl morpholine	0.40%	Before sterilization	Inhibiting HMGR	Bhosale, (2004)

The combination mode and concentrations of combined additives are shown in Table 2. Eighteen different combinations of additives include 15 combinations of two regulators, a combination of four regulators, and two combinations of five regulators.

**TABLE 2:** Combined addition of metabolic regulators.

Metabolic regulators	Added concentration
Glutamic acid + sodium gluconate	3 g/L + 1 g/L
Glutamic acid + citric acid	3 g/L + 2 g/L
Glutamic acid + penicillin	3 g/L + 1 mg/L
Glutamic acid + ethanol	3 g/L + 1 g/L
Glutamic acid + triclosan	3 g/L + 5 mg/L
Glutamic acid + fluconazol	3 g/L + 120 mg/L
Pyruvic acid + sodium gluconate	2 g/L + 1 g/L
Pyruvic acid + citric acid	2 g/L + 2 g/L
Pyruvic acid + penicillin	2 g/L + 1 mg/L
Pyruvic acid + ethanol	2 g/L + 1 g/L
Pyruvic acid + triclosan	2 g/L + 5 mg/L
Pyruvic acid + fluconazol	2 g/L + 120 mg/L
Glutamic acid + pyruvic acid	3 g/L + 2 g/L
Penicillin + ethanol	1 mg/L + 1 g/L
Triclosan + fluconazol	5 mg/L + 120 mg/L
Penicillin + ethanol + triclosan + fluconazol	1 mg/L + 1 g/L + 5 mg/L + 120 mg/L
Glutamic acid + penicillin + ethanol + triclosan + fluconazol	3 g/L + 1 mg/L + 1 g/L + 5 mg/L + 120 mg/L
Pyruvic acid + penicillin + ethanol + triclosan + fluconazol	2 g/L + 1 mg/L + 1 g/L + 5 mg/L +1 20 mg/L

### Determination of Carotenoid Content

Dry weight method was used in the analysis. An appropriate volume of fermentation broth was centrifuged for 5 min at 4,000 rpm, and the yeast was washed twice with distilled water and dried to constant weight at 105°C.

Astaxanthin in the samples was detected by spectrophotometry. DMSO method was used to break the walls (Sedmak et al., 1990; Ni et al., 2008). The fermentation broth (1 ml) was collected and centrifuged, and the pellet was washed twice with distilled water and added with 2 ml of DMSO preheated to 75°C. Ethanol (5 ml) was added, shaken, and centrifuged at 4,000 rpm for 5 min to obtain the supernatant, which was fixed to 10 ml with ethanol. The absorbance values were examined by UV spectrophotometry (Ni et al., 2005) at a wavelength of 474 nm. An astaxanthin standard curve was constructed, and the astaxanthin content was calculated according to the standard curve equation.

### Fluorescence Quantitative PCR, Fatty Acid and Isopentenyl Pyrophosphate Determination

The 120 h fermentation broth was taken and 18 s was used as the internal reference gene to perform fluorescent quantitative PCR. Quantitative PCR instrument ABI7300 and fluorescent quantitative PCR kit were used to carry out relative quantification of target gene.

The method of Li et al. (2018) was referred to determine fatty acid content. The method of Schaefer et al. (2021) was used to determine isopentenyl pyrophosphate content.

### Data Processing

The experimental data in this article had at least three biological repetitions. Sigma Plot version 14.0 (Systat Software Inc., San Jose, California, United States) was used to analyze the data and make charts. SPSS version 20 (International Business Machines Corporation, Armonk, New York State, United States) was used in variance analysis. PPT version 16051 (Microsoft, Redmond, Washington, United States) and ChemDraw version 19 (PerkinElmer, Inc., Waltham, Massachusetts, United States) were used to draw pictures.

## Results and Discussion

### Effects of Single Metabolic Regulators on *P. rhodozyma* Biomass

**FIGURE 1:**
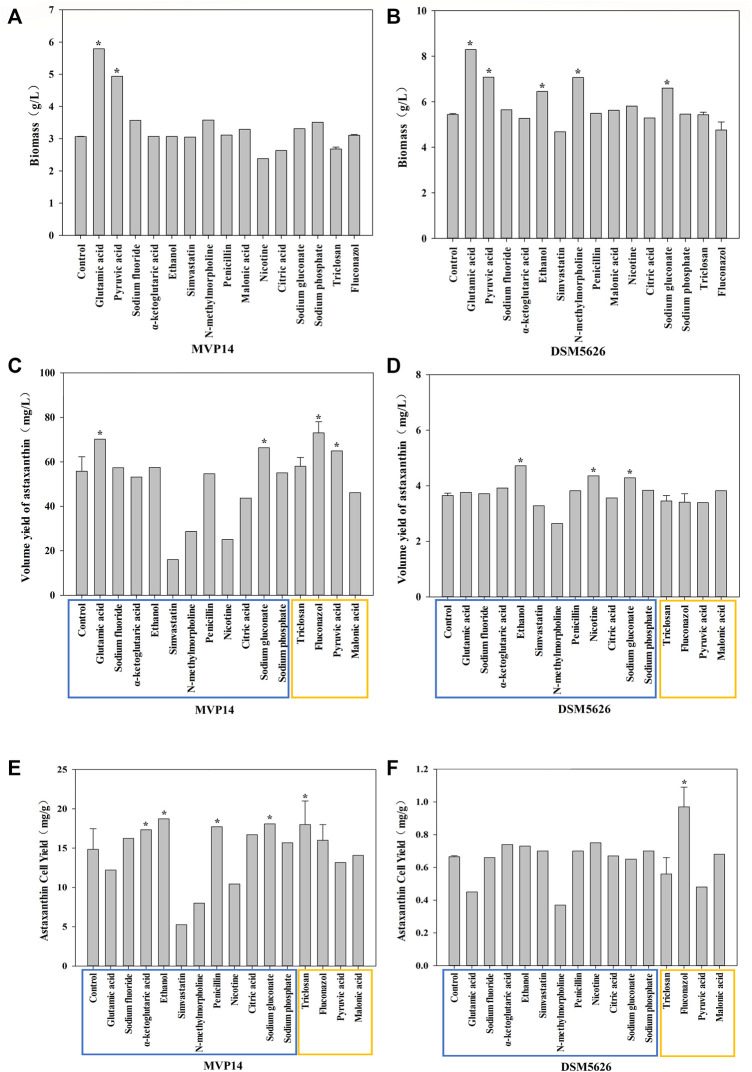
The effect of single regulator on biomass of *P. rhodozyma* MVP14 **(A)** and DSM5626 **(B)**. The effect of single regulators on astaxanthin volume yield of *P. rhodozyma* MVP14 **(C)** and DSM5626 **(D)**. The effect of single regulators on astaxanthin cell yield of *P. rhodozyma* MVP14 **(E)** and DSM5626 **(F)**. The blue box shows the additives acting on the synthetic pathway, and the yellow box shows the additives acting on the competitive pathway. (* significantly different from control, *p* < 0.05).

The biomass of *P. rhodozyma* is closely regulated to the accumulation of metabolites, such as 2-ketoglutarate and glyoxylic acid (Wang, B. et al., 2019). Glutamic acid, as a nitrogen source, promotes cell growth (Albers et al., 1996). Thus, the addition of glutamic acid can increase the biomass of *P. rhodozyma*. Pyruvate is an important node biomolecule for acetyl-coenzyme A (CoA) that can directly increase the substrate required for growth and thus increase biomass (Lu et al., 2019). The effects of single metabolic regulators on the biomass of *P. rhodozyma* MVP14 and DSM5626 are shown in Figure 1. Glutamic acid was the most prominent among the tested regulators in increasing the biomass of *P. rhodozyma* MVP14 and DSM5626, by 89 and 52.4%, respectively. In addition, pyruvic acid remarkably increased the biomass of *P. rhodozyma* MVP14 and DSM5626 by 61 and 30.1%, respectively. The other additives did not have a remarkable effect on *P. rhodozyma* biomass. These results are consistent with the findings of previous studies (Lan et al., 2002; Lu et al., 2019). Therefore, EMP and HMP were found to be positively correlated with *P. rhodozyma* biomass.

### Effects of Single Metabolic Regulators on the Volumetric Yield of Astaxanthin

Four additives, fluconazol, glutamic acid, sodium gluconate, and pyruvic acid, significantly increased the volume yield of astaxanthin in *P. rhodozyma* MVP14 by 30.9, 25.8, 18.9, and 16.4%, respectively, while three additives, ethanol, nicotine, and sodium gluconate, significantly increased the volume yield of astaxanthin in *P. rhodozyma* DSM5626 by 29.3, 19.5, and 17.5%, respectively. Fluconazol has been reported to inhibit the ergosterol synthesis pathway (Yang et al., 2018), which is a competitive pathway for astaxanthin synthesis (Miao et al., 2011). Some studies have reported that adding low-concentration ethanol can increase β-hydroxy-β-methylglutaryl-CoA reductase (HMGR) activity and promote astaxanthin synthesis (Gu et al., 1997; Xiao et al., 2015), which could explain the observed increase in volume yield of astaxanthin. The FAS pathway is a competitive pathway for pigment synthesis. When this pathway is weakened, carbon flow is increased to promote pigment synthesis and astaxanthin production (Miao et al., 2011). Astaxanthin volume yield can be increased by improving biomass and inhibiting competition pathways, such as the ergosterol synthetic and FAS pathways (Cheng et al., 2016; Yang et al., 2018). The astaxanthin volumetric yields of *P. rhodozyma* MVP14 and DSM5626 are plotted in Figure 1. Glutamic acid and fluconazol improved the volume yield of astaxanthin in the high-yield strain MVP14, by 25.8 and 30.9%, respectively (Figure 1C). The increase in volume yield of astaxanthin by glutamic acid might be due to the effect of glutamic acid on cell biomass (Figure 1A). Meanwhile, fluconazol increased the volume yield of astaxanthin by increasing the carbon flux of the MVA pathway involved in astaxanthin synthesis. The volumetric yields in MVP14 and DSM5626 increased by 3 and 29.3%, respectively, with the addition of ethanol. Moreover, triclosan, an inhibitor of FAS, also increased the astaxanthin volume yield in MVP14 by 4%, but did not have an effect in DSM5626.

**FIGURE 2:**
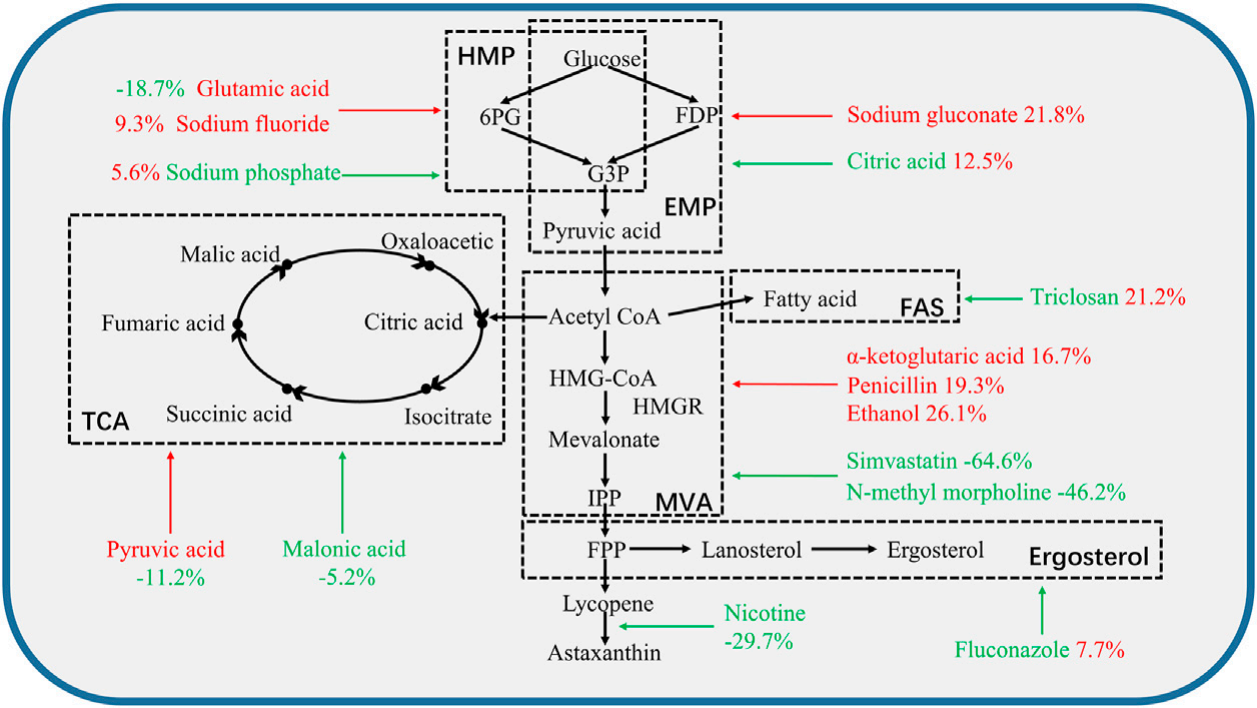
The effect of single regulator on the metabolic pathway in *P. rhodozyma* MVP14. The black dashed lines frame the corresponding metabolic pathways described in black bold text. The red additives represent the promoters of this pathway or enzyme. Green additives represent inhibitors of this pathway or enzyme. Red numbers mean an increase in astaxanthin cell yield. Green numbers mean reduction of astaxanthin content. The arrow points to the pathway or site affected by the corresponding additive. HMP: hexose monophosphate pathway, EMP: Embden-Meyerhof-Parnas pathway, TCA: tricarboxylic acid cycle, MVA: mevalonate pathway, FAS: fatty acids pathway, 6PG: 6-phosphogluconate, FDP: fructose-1, 6-diphosphate, G3P: glucose-3-phosphate, IPP: isopentenyl pyrophosphate, FPP: farnesyl pyrophosphate.

Ethanol and nicotine were the most effective in increasing the astaxanthin yield in DSM5626 (Figure 2), while glutamic acid and fluconazol were the most effective in increasing the astaxanthin yield in MVP14 (70.15 and 73 mg/L, respectively) (Figure 2). Among the regulators which inhibit the competition pathways, fluconazol was the most effective and increased the yield by 30.9% in MVP14. Whereas, glutamic acid was the most effective regulator that promoted the synthetic pathways and increased the volume yield by 25.8% in MVP14. These results are consistent with the findings of previous studies (Gu et al., 1997; Miao et al., 2011; Xiao et al., 2015; Cheng et al., 2016; Yang et al., 2018). Therefore, inhibition of the FAS and ergosterol synthesis pathways, and the promotion of HMGR contributed to the enhancement of astaxanthin yield.

### Effects of Single Metabolic Regulators on the Astaxanthin Cell Yield of *P. rhodozyma*

Astaxanthin cell yield, also known as astaxanthin content, refers to the astaxanthin production capacity of a single cell. Ethanol can promote the enzymatic activity of HMGR (Gu et al., 1997), which is an essential enzyme in the MVA pathway for astaxanthin synthesis (Xiao et al., 2015). Sodium gluconate can enhance EMP (Wushensky et al., 2018), which can provide the substrate acetyl-CoA to promote astaxanthin synthesis and increase astaxanthin cell yield. Improving astaxanthin cell yield is the primary focus in industrial production of astaxanthin. The addition of ethanol, sodium gluconate, and triclosan increased the astaxanthin cell yield in *P. rhodozyma* MVP14 by 26.1, 21.8, and 21.2%, respectively (Figure 1). In addition, nicotine can also increase the astaxanthin content in *P. rhodozyma* DSM5626 by 12.8% (Figure 1). These results further indicated that the inhibition of competition pathways, such as the FAS pathway, had a remarkable effect on astaxanthin synthesis (Cheng et al., 2016). The addition of these three additives had the most remarkable effect on astaxanthin production in *P. rhodozyma*. These results are consistent with the findings of previous studies (Gu et al., 1997; Xiao et al., 2015). The promotion of the EMP and MVA pathways contributed to the improvement in astaxanthin cellular yield.

The effect of a single regulator of astaxanthin cell yield on the metabolic pathway in *P. rhodozyma* MVP14 is shown in Figure 2. The HMP pathway is the initial pathway in astaxanthin biosynthesis that initiates conversion of glucose to astaxanthin (Pan et al., 2020). Glutamic acid promoted the HMP pathway, but reduced the astaxanthin cell yield by 18.7%, as glutamic acid promotes the use of carbon sources for biomass growth and NADPH synthesis (An, 2001). Sodium phosphate can inhibit the HMP pathway, but can also promote the conversion of glucose to EMP (Liu et al., 2022); therefore, it can increase astaxanthin production by 5.6%. The addition of citric acid increased astaxanthin cell yield by 12.5%, as citric acid inhibits the EMP pathway, provides raw materials for the TCA cycle, and reduces the entry of acetyl-CoA into TCA cycle (Li et al., 2012).

After completion of the EMP pathway, the metabolites entered the MVA pathway. Ethanol and penicillin promoted MVA synthesis and improved astaxanthin cell yield by 26 and 19.3%, respectively, whereas simvastatin and N-methyl morpholine inhibited MVA synthesis and decreased astaxanthin cell yield by 64.6 and 46.2%, respectively. The FAS and ergosterol synthesis pathways are the competing pathways for astaxanthin synthesis (Pan et al., 2020). Fluconazol inhibited the ergosterol pathway and increased astaxanthin production by 7.7%. Triclosan increased astaxanthin production by 21.2% by inhibiting the FAS pathway.

Ethanol and triclosan showed the best results among the promoters of the synthesis pathways and inhibitors of competition pathways, respectively. This result suggests that inhibition of the FAS pathway is of great importance for regulation of competing pathways. The improvement of astaxanthin cell yield by a single regulatory agent was limited up to 5.6–26.1%, which could still be improved.

### Effects of Different Regulator Combinations on the Astaxanthin Production of *P. rhodozyma*

**FIGURE 3:**
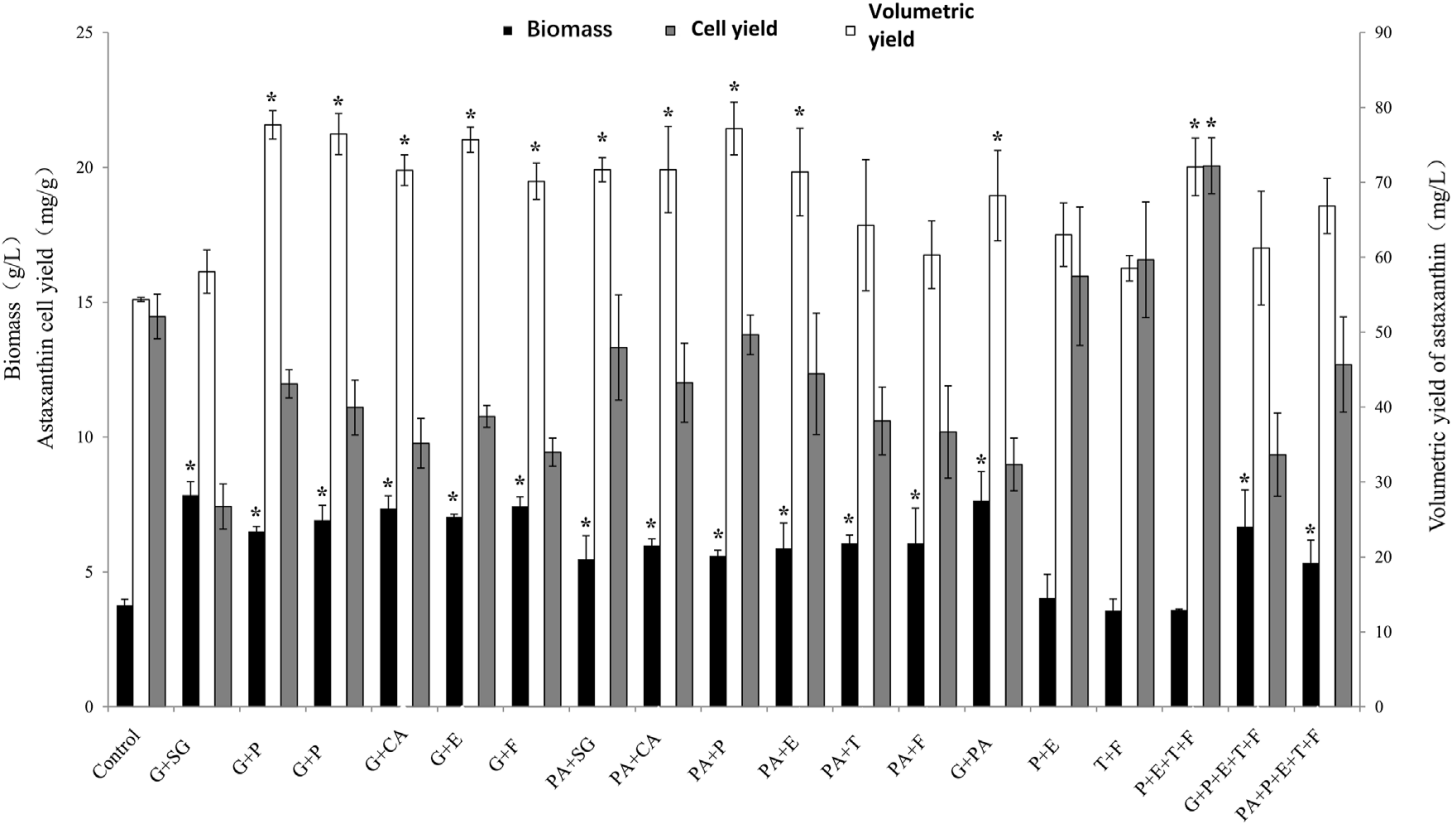
The effect of different regulator combinations on biomass, astaxanthin cell yield and volumetric yield in *P. rhodozyma* MVP14. The abbreviation of abscissa in the figure is the following meaning, G: Glutamic acid, SG: Sodium gluconate, CA: Citric acid, P: Penicillin, E: ethanol, T: Triclosan, F: Fluconazol, PA: Pyruvic acid. (* significantly different from control, *p* < 0.05).

Combinatorial regulation is a beneficial modification strategy that simultaneously targets multiple metabolic nodes and has been successfully applied in other microorganisms (Li et al., 2012; Chaturvedi et al., 2021). When metabolic regulators are combined in the fermentation process, multiple nodes of the synthesis process can be simultaneously regulated to increase the regulation efficiency. *P. rhodozyma* JMU-MVP14, an astaxanthin overproducing strain, was selected to further optimise production using combinations of the regulators. Based on the different effects of the single metabolic regulators, glutamate, pyruvate, sodium gluconate, citric acid, penicillin, ethanol, triclosan, and fluconazol were selected for the combination treatments, as shown in Figure 3.

Glutamic acid with penicillin, pyruvic acid with penicillin, glutamic acid with citric acid, and glutamic acid with triclosan increased the astaxanthin volume yield by 42.8, 42, 40.6, and 39.2%, respectively. The effect of the combination treatments was higher than the effect of the individual regulators. This suggests that their promotive effects were synergistically enhanced. Biomass and astaxanthin volume yields were increased by different combinations of glutamic acid or pyruvic acid compared to the control group. Glutamic acid was more effective than pyruvic acid in promoting the growth of the JMU-MVP14 strain, which is consistent with the results of the single regulators. The combination of glutamic acid and pyruvic acid further enhanced the growth of JMU-MVP14. This result suggests that the growth-promoting effects of the regulators can be further enhanced when the regulators are used in combination. In addition, the combination of glutamic acid and sodium gluconate had an obvious synergistic effect in promoting biomass growth and astaxanthin volumetric yield. Triclosan with fluconazol and penicillin with ethanol increased astaxanthin cell yield by 25 and 15.7%, respectively. The combination of penicillin, ethanol, triclosan, and fluconazol remarkably increased astaxanthin cell yield by 51%, reaching 22 mg/g. These results suggest that the combined effect of the four additives on cell yield was better than that of the combination of two additives. This showed that there was still the possibility of synergy and overlap between the combined additives.

**FIGURE 4:**
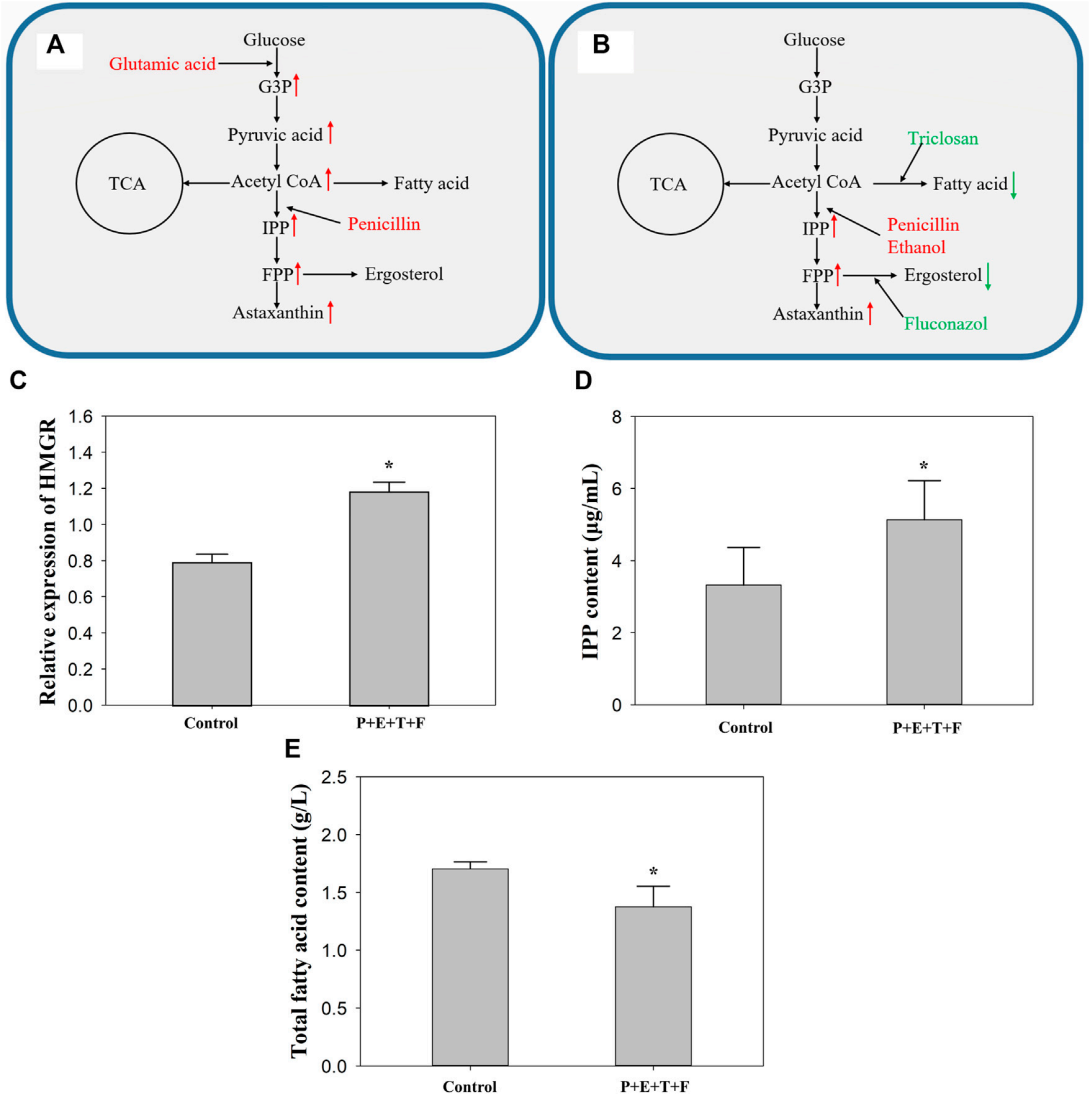
The effect of different regulator combinations on the metabolic pathway in *P. rhodozyma* MVP14. **(A)** Highest astaxanthin volumetric yield with adding glutamic acid and penicillin. **(B)** Highest astaxanthin cell yield with adding penicillin, ethanol, triclosan and fluconazol. Red substance represents the promotion of current pathway, while green substance represents the inhibition. Changes in the relative expression of HMGR gene **(C)**, IPP content **(D)** and total fatty acids **(E)** after adding penicillin, ethanol, triclosan and fluconazol to *P. rhodozyma* MVP14. The red arrow indicates that the metabolite is increased, and the green one indicates that it is decreased. G3P: glucose-3-phosphate, IPP: isopentenyl pyrophosphate, FPP: farnesyl pyrophosphate, TCA: tricarboxylic acid cycle, P: penicillin, E: ethanol, T: triclosan, F: fluconazol. (* significantly different from control, *p* < 0.05).

We compared the effects of the combination with the highest astaxanthin volumetric yield with that of the combination with the highest astaxanthin cell yield. The metabolic nodes affected by the combined additives were mapped to understand how the combined additives affected the metabolic pathway (Figure 4). As shown in Figure 4A, glutamic acid promoted glyceraldehyde-3-phosphate synthesis, while penicillin promoted isopentenyl pyrophosphate synthesis. Both compounds are intermediate products of astaxanthin biosynthesis. Therefore, glutamic acid and penicillin simultaneously promoted astaxanthin synthesis by increasing volumetric yield. However, this combination also decreased astaxanthin cell yield as it enabled the use of a partial flux to increase biomass.

As shown in Figure 4B, penicillin with ethanol had a better regulatory effect and synergistic promotion effect than either penicillin or ethanol alone. Penicillin and ethanol have been reported to promote HMGR and MVA kinase in the astaxanthin synthetic pathway (Gu et al., 1997; Wang et al., 2012). Untreated MVP14 was used as the control group to determine the relative expression level of HMGR gene in *P. rhodozyma* with penicillin, triclosan, fluconazol, and ethanol (Figure 4C). The relative expression of HMGR after addition of the combination increased by 49.0%. The IPP content in the MVA pathway was also measured, and the content increased by 54.8% after the addition of the combined additives (Figure 4D). It is showed that HMGR, an important synthetic enzyme in the MVA pathway, increases carotenoid production when its expression increases (Loto et al., 2012). Ergosterol is a branch point in the MVA pathway that can affect HMGR through a negative feedback mechanism (Loto et al., 2012). Therefore, ergosterol inhibition could promote astaxanthin production by inhibiting the competing pathway and enhancing HMGR expression in the synthetic pathway. Moreover, fluconazol with triclosan can inhibit the competing pathways of astaxanthin synthesis, FAS and ergosterol synthesis pathways (Cheng et al., 2016; Sanglard et al., 1998). Compared to the control group, the total fatty acid content of *P. rhodozyma* decreased by 19.3% after inhibition of the FAS pathway (Figure 4E). In general, fungi possess a very active acetyl-CoA metabolism; carotenoids and fatty acids require acetyl-CoA as a synthetic precursor (Sandmann et al., 2021). Fatty acid accumulation is 1000-fold higher than that of carotenoids in *P. rhodozyma* (Miao et al., 2010), which indicates that most acetyl-CoA is used for lipid synthesis. The inhibition of the FAS pathway is therefore expected to contribute considerably to the astaxanthin synthesis pathway, including the MVA pathway. The astaxanthin content of both high- and low-yield strains increased with the addition of fluconazol and inhibition of FAS. The competing pathways were inhibited, and the synthetic pathway was enhanced; the synergistic effect in the two directions promoted astaxanthin synthesis. This combination increased both astaxanthin cell yield and volumetric yield. A comparison of Figures 1, 4A revealed that merely promoting and modulating the master pathway is not sufficient to improve astaxanthin cell yield. The promotion of master pathways should be combined with the inhibition of competing pathways to effectively increase astaxanthin cell yield. As shown in Figure 3, the combination of some regulators can increase the biomass but decrease the astaxanthin cell yield. Therefore, we speculated that if further modulation of the cell yield is necessary, a regulator that stimulates biomass growth should not be used in the combination. Therefore, when increasing cell yield, the stimulation of biomass growth should be avoided, and regulation of the main synthesis and competition pathways of astaxanthin should be the focus. This study provides theoretical support for subsequent research on genetic pathway modifications.

## Conclusion

Fifteen single regulators of six metabolic pathways were screened to improve astaxanthin production in *P. rhodozyma* MVP14 and DSM5626. Glutamic acid and fluconazol were the most effective additives at improving the astaxanthin volume production in MVP14, improving volumetric yield by 30.9 and 25.8%, respectively. Ethanol was the most effective in increasing astaxanthin production in DSM626, by 29.3%. Six effective additives were screened as combination additives. We found that although glutamic acid and penicillin simultaneously promoted the astaxanthin synthesis pathway for volumetric yield, the combination caused a decrease in astaxanthin production. The optimal combination to increase the cell yield of astaxanthin was penicillin, ethanol, triclosan, and fluconazol, with an increase of 51%, reaching 22.4 mg/g. In this combination, triclosan and fluconazol inhibits the competitive FAS and ergosterol pathways to increase astaxanthin cell yield. This was in synergy with the promotion of HMGR and MVA in the astaxanthin synthetic pathway by penicillin and ethanol. Our results suggest that promotion of master pathways should be combined with inhibition of competing pathways to effectively increase astaxanthin cell yield. In addition, stimulation of the biomass pathway should be avoided. This study provides theoretical support for subsequent research on the genetic modification of the astaxanthin synthesis pathway.

## Data Availability

The original contributions presented in the study are included in the article/Supplementary Material, further inquiries can be directed to the corresponding author.
